# Measurement and Quality Improvement

**DOI:** 10.1097/pq9.0000000000000886

**Published:** 2026-09-28

**Authors:** Lloyd P. Provost

**Affiliations:** 1 From the Associates in Process Improvement, Austin, Tex.

The article in this issue, *Accuracy of Various Methods for the Surveillance of Hospital Acquired Venous Thromboembolism in a Children’s Hospital* by Huibonhoa et al,^1^ focuses on measurement—specifically, methods for detecting hospital-acquired venous thromboembolism (HA-VTE). At first glance, readers may ask why an article centered on measurement rather than an intervention appears in a healthcare quality and safety improvement journal. The answer is that meaningful improvement depends on the ability to measure outcomes accurately and consistently.

A phrase often used in quality improvement (QI) discussions captures this idea: “You can’t fatten a cow by weighing it.” The saying reminds us that measurement alone does not produce improvement. However, without measurement, it is extremely difficult to determine whether changes in care lead to better outcomes. Measurement does not create improvement, but it makes improvement visible and learnable.

Leaders in improvement science have long emphasized this point. Shewhart^2^ and Deming^3^ both argued that improvement requires a stable and credible measurement system. Without reliable data, organizations cannot distinguish real change from random variation, learn from interventions, or sustain improvement over time.

In his landmark 1931 book *Economic Control of Quality of Manufactured Product*, Shewhart devoted an entire chapter to measurement.^2^ He wrote: “In any program of control, we must start with observed data; yet data may be either good, bad, or indifferent. Of what value is the theory of control if the observed data going into that theory are bad?” His point remains highly relevant in modern healthcare improvement efforts.

Deming^3^ later reinforced this principle in *Out of the Crisis*, emphasizing that “statistical control of the process of measurement is vital; otherwise, there is no meaningful measurement.” If the measurement process itself is unstable or inaccurate, conclusions about improvement will inevitably be unreliable.

Measurement has therefore always played a central role in the QI movement. Since 1968, the American Society for Quality has maintained a Measurement Division devoted to advancing knowledge about measurement systems and their role in quality.^4^ Most modern texts on improvement science include dedicated discussions of measurement, recognizing that improvement requires not only changes in care processes but also confidence in how results are assessed.^5^

In practical terms, measurement in improvement projects typically includes several complementary types of metrics:

- Outcome measures—indicators of how the system is performing and the results experienced by patients.
- Process measures—indicators of whether key steps in care are being performed as intended.
- Balancing measures—metrics used to detect unintended consequences or impacts on other parts of the system.
- Plan, Do, Study, Act (PDSA) measures—measures used within specific PDSA cycles to evaluate whether a tested change is producing the expected effect.

Each of these measures depends on an underlying measurement process. In modern health systems, these processes often involve automated extraction of data from electronic health records. Because the data are readily available, the measurement processes themselves are sometimes taken for granted.

The study by Huibonhoa et al highlights why this assumption can be problematic. Many healthcare organizations rely on administrative data—such as diagnostic codes—to detect adverse events such as HA-VTE. Yet such sources frequently provide incomplete or inaccurate surveillance.

The authors systematically compare several automated rule-based detection approaches, including administrative codes, ultrasound imaging reports, medication orders, and other clinical triggers. Their analysis demonstrates that each method has distinct strengths and limitations. Some approaches identify a large proportion of true cases but generate many false positives, whereas others are more specific but miss a substantial number of events.

The study shows that relying on any single data source can lead to substantial misclassification. For improvement teams attempting to monitor patient safety, such measurement error can obscure true system performance.

These findings have important implications for QI practice. Improvement initiatives often depend on automated data extraction from electronic health records to support timely monitoring. If the underlying detection rules are poorly specified, however, the resulting measures may misrepresent actual performance. Organizations may incorrectly conclude that an intervention is successful—or failing—based not on changes in care, but on artifacts of measurement.

By rigorously evaluating multiple detection strategies and demonstrating the value of combining rule-based approaches, the authors provide a practical framework for developing more reliable surveillance systems. Their work strengthens the measurement infrastructure needed to monitor HA-VTE and other safety outcomes.

More broadly, the study illustrates a principle that extends far beyond VTE surveillance. Many hospital improvement initiatives rely on outcome measures derived from administrative or electronic data sources. Before these measures are used to guide decisions or evaluate improvement efforts, organizations must understand the accuracy and limitations of the underlying detection methods. Studies that evaluate measurement systems, therefore, play an essential role in enabling effective improvement.

This article is important because it addresses the measurement foundation required to improve hospital safety. Specifically, it

- demonstrates the limitations of commonly used administrative data for event detection;
- quantifies the performance of multiple automated surveillance methods;
- shows that combining detection strategies improves case identification;
- supports the development of automated monitoring for HA-VTE;
- enables more reliable use of statistical process control in improvement work.

Although the article is not a traditional intervention study, it provides essential infrastructure for improvement. Without such work, efforts to reduce hospital-acquired harm might be guided by unreliable signals. By improving the accuracy of our detection and monitoring of HA-VTE, Huibonhoa et al provide a stronger measurement foundation for learning and improvement—an essential step toward reducing preventable harm in hospitalized patients.

Bottom line: We should expect that most articles in this journal should be classical improvement initiatives with a clear aim, changes, and measures. However, there is also a place for studies on improving the processes we use to create these measures.
